# A Proposal to Improve the Effectiveness of the Deflection Control Method Provided by Eurocodes for Concrete, Timber, and Composite Slabs

**DOI:** 10.3390/ma14247627

**Published:** 2021-12-11

**Authors:** Tommaso D’Antino, Marco Andrea Pisani

**Affiliations:** Department of Architecture, Built Environment and Construction Engineering, Politecnico di Milano, 20133 Milan, Italy; marcoandrea.pisani@polimi.it

**Keywords:** deflection control, curvature, serviceability, reinforced concrete, timber, composite slab

## Abstract

Limited deflection of structural members represents an important requirement to guarantee proper functionality and appearance of building and infrastructures. According to Eurocodes, this requirement is ensured by limiting the maximum deflection of horizontal structural members to a fraction of their span. However, each Eurocode provides different maximum deflection limits, which are independent of the type of superstructures considered. Thus, the respect of these limits may not always guarantee the integrity of certain superstructures. In this paper, the reliability of the Eurocode deflection control methods, in guaranteeing the integrity of the superstructures, is assessed and discussed. First, different types of horizontal member, namely rib and clay (hollow) pot, composite steel–concrete, and timber beam slabs are designed to respect the deflection limit enforced by the Eurocodes. Then, the maximum curvature developed by these members is compared with the ultimate (limit) curvatures of various superstructures (e.g., ceramic and stone tile floorings). The results obtained show that the approach adopted by Eurocode 2 may provide non-conservative results, but also that the rules proposed by Eurocodes 4 and 5, albeit more reliable, do not always guarantee the integrity of the superstructure. Based on these results, an alternative method, based on the curvature control, is proposed and its advantages and limitations critically discussed. This method appears simpler and more reliable than the method currently adopted by the Eurocodes.

## 1. Introduction

Deflection control is crucial to guarantee proper functionality and good appearance under service loads of buildings and infrastructures [1,2,3]. However, the computation of the short- and long-term maximum deflection of a horizontal structural member can be a cumbersome task, and simplified analysis and verifications are often employed [4]. Although excessive deflection does not impair the structural safety, it seriously affects the serviceability of the structure. Cracking of the floor, due to the excessive deformability of the supporting slab, is a well-known issue, although often underestimated. Usually, cracking is attributed to the building settling, which may be responsible for the opening and widening of cracks over time, whereas the possibility that cracking is due to excessive slab deformability is rarely taken into account. Cracking caused by structure settling and slab deformability can be easily distinguished. In the former case, cracks occur and keep opening until the settling is complete. Afterwards, their width remains constant. In the latter case, the crack width varies with varying the service load (thus, the slab deflection). Therefore, while cracking produced by the structure settling can be repaired once the phenomenon is exhausted, cracking due to the excessive deformability, if repaired, will reoccur, since it is the result of a congenital deficiency of the structural element.

Controlling the maximum deflection is particularly hard in the case of RC structures, where the occurrence of concrete cracking and presence of concrete creep affects both the short- and long-time deflection of horizontal members. Since the limit values defined by Eurocode 2 [4] do not depend on the type of superstructures or finishes (including their application method [5]), they can provide non-reliable results, especially when stiff and brittle elements, such as ceramic or stone tiles (a quite common solution in Mediterranean countries), are used as floor finishing.

Although a proper computation of the member vertical deflection is quite difficult, the approaches currently provided by European standards are rather simplified. In general, deflection control is performed by enforcing a limit to the vertical displacement of horizontal members. This limit should depend on the intended use of the structure (residential, office, etc.), finishes, and superstructures (e.g., partitions). Eurocode 2 [4], Eurocode 4 [6], and Eurocode 5 [7], which are the European design and verification codes for reinforced concrete (RC), composite steel–concrete, and timber structures, respectively, limit the vertical displacement of horizontal members to a fraction of the associated span. However, each Eurocode provides a specific definition of the limiting deflection, and significant inhomogeneity can be found among these limiting values. This differentiation does not seem justified, considering that variable service loads, superstructures, and finishes could be the same, regardless of the type of structure.

This paper aims at critically reviewing the approaches provided by Eurocode 2 [4], Eurocode 4 [6], and Eurocode 5 [7], which are derived from ISO 4356 [8] (as stated by Eurocode 2), for the control of horizontal members deflection. The limits of these approaches and discrepancies among them are pointed out, and a new approach, that appears simpler than those provided by the Eurocodes, is proposed. To do so, some horizontal member types typical of the Mediterranean Basin and Alpine region, and of some countries in Latin America, are first designed according to the Eurocode indications. These horizontal members are:

- rib (clay pot or hollow block) slab;
- composite steel–concrete slab;
- traditional slab made of timber beams and planks.

Subsequently, the deformability of the following types of superstructures is studied, computing the limit curvature value associated with the absence of damage (cracking) in the following elements:

- partition walls;
- floorings (of various type).

These curvature values are then compared with those obtained by applying the maximum service load, i.e., the characteristic load combination [9], to the horizontal members designed according to the Eurocodes. The analysis focuses on horizontal members and superstructures frequently adopted in areas where Eurocodes apply. However, the same analysis could be extended to other structural systems, including innovative solutions, such as fiber-reinforced polymer (FRP) reinforced concrete members, for which the design process is controlled by the member deformability [10].

Comparison between curvature values, obtained by enforcing the limits imposed by the Eurocodes and corresponding superstructure limit curvatures, showed non-conservative results in some cases, which proves that Eurocode provisions do not always guarantee the integrity of the most rigid finishing elements. A new curvature control method to verify the horizontal member deflection under service loads is finally proposed and discussed. This method represents a performance approach, with some important advantages with respect to the approach currently adopted by Eurocodes, as discussed below.

## 2. Bending Limit Imposed by Superstructures and Finishes

A reliable deflection control method should provide deflection limit values capable of guaranteeing the absence of damage (cracking) in the superstructures. Since these limits vary depending on the type of superstructure, a separate analysis of the deflection limits associated to partition walls and various types of flooring was performed in this study. Representative geometrical and mechanical properties of the superstructures considered were collected from available scientific literature and product technical sheets.

The behavior of ceiling finishes, among which the most brittle is the plaster finishing applied to ribbed concrete slabs [11], was not investigated because damage of ceiling plasters is prevented by limiting the slab crack opening through crack control.

### 2.1. Partition Walls

Several different alternatives, such as plasterboard walls or walls made of cellular concrete blocks, can be adopted to realize partition walls. Among all these possible solutions, hollow clay brick walls covered with plaster appear to be the most brittle solution (see, for instance, [12,13,14]). Although cracking of partition walls represents an important issue, when caused by excessive deformability of the supporting slab is usually preceded by cracking of the tiles, which is often associated with a slab curvature lower than that associated with cracking of the partitions. Indeed, partition walls have a significant strength when loaded in their mean plane and are able to compensate for limited differential settlements through the arch effect [15]. Furthermore, partition walls are often placed in the same positions in the different stories of a building, which limits the deflection of the slab in these positions, thus preventing the partition cracking.

It should be noted that facade walls do not usually have important deflection problems because they are supported by the perimeter beams or are themselves structural elements, as in the case of masonry buildings.

### 2.2. Floorings

Floorings can be made with many different materials. Among them, the most diffused are:

- carpet flooring (moquette);
- synthetic materials (usually rubber, PVC, or linoleum);
- resin;
- timber;
- stone (e.g., marble, granite, or sandstone);
- ceramic.

(i) Three main types of carpet can be identified, depending on the type of fabric (namely natural, polyamide, and polyester fiber fabric [16]). However, since the mechanical behavior of carpets is strictly related to the support to which the fabric is applied, and since this support is generally a synthetic material (usually a rubber), the same considerations regarding synthetic materials can be applied to carpets.

(ii) Rubber, PVC, or linoleum floorings have high deformability (see for instance [17]) and, therefore, are able to adapt to deformations and cracks of the slab.

(iii) Epoxy resins are generally employed for floorings [18]. These resins have good strength and high deformability, although their flexural strength decreases with increasing thickness. The values supplied by various manufacturers suggest that the thickness varies between 1.5 and 3 mm, the flexural strength between 25 and 60 MPa, the elastic modulus between 2800 and 4200 MPa, and the elongation at break between 7.5 and 8% [19,20]. These values allow the flooring to withstand significant flexural deformations of the slab.

(iv) Timber floors may be nailed or glued to the subfloor. In addition, floating timber flooring (i.e., flooring laid on an underlay that provides good noise insulation) can be found. The first two solutions are the most sensitive to bending of the slab because the timber floor is directly and firmly connected to it. Various types of timber board, which differ for geometry, color, and species, can be used in timber floorings. Among the various species available, one of the most employed is oak, which offers excellent mechanical properties and relatively low cost. Indeed, its elastic modulus is approximately 12,500 MPa, while the bending strength can reach 108 MPa (in the absence of defects), although these values may vary with ambient temperature and relative humidity [21]. The thickness of the boards generally varies between 10 and 22 mm, with the width between 70 and 250 mm and length between 250 and 2500 mm. Due to its elastic modulus, this type of flooring is able to adapt to deformations far greater than those generally considered acceptable for the slabs of a multi-story building [22].

(v) The bending strength of stone tiles depends on the thickness to width ratio, as well as on the type of stone [23]. The quality of these natural products, which is the result of physical (e.g., porosity) and mechanical properties of the stone, can vary significantly even for blocks extracted from the same quarry [24]. Furthermore, stone materials have a brittle behavior [25], which makes stone floorings particularly sensitive to bending of the slab. Floorings made by granite and marble tiles are among the most diffused solutions. In these cases, the tile thickness may vary between 10 and 30 mm, regardless of the size, which may vary from 50 × 50 mm^2^ to 600 × 1200 mm^2^. The minimum bending strength found in the literature for natural stones is 18 MPa for marble and 20 MPa for granite [26,27]. These values increase up to 64 MPa, both for marble and granite, when artificial stones are employed [28]. Artificial stones are made industrially starting from the same precursor materials of the corresponding natural stones and have a similar aspect and geometry (thickness and size) [23,29].

(vi) Ceramic tiles are subjected to the provisions of EN 14411 [30], which enforces specific characteristics and refers to EN ISO 10545-4 [31] for the determination of the tile flexural strength by means of a three-point bending test. Tiles can have very different sizes, with thickness up to 20 mm and size up to 1200 × 2400 mm^2^. The behavior of ceramic tiles is always elastic-brittle and depends on their width [32,33]. The determination of their strength is complicated by the fact that many producers simply state that their ceramic tiles exceed the minimum value imposed by EN ISO 10545-4 [31]. However, bending strength values varying between a maximum of 55 MPa and minimum of 35 MPa, which can reduce to 15 MPa in the case of small-size tiles (thickness less than 15 mm and size not exceeding 200 × 250 mm^2^) can be found on the market [28,34,35,36,37].

## 3. Limit Curvature Values

The limit curvature of a material subjected to bending is the curvature value associated with the attainment of the material tensile strength, i.e., with the occurrence of the first crack. Available standards and design guidelines provide criteria for guaranteeing absence of damage in brittle floorings [38]. In this paper, the limit curvatures of ceramic, marble, and granite tiles were considered, as these flooring types are brittle and largely used in residential and commercial buildings. For each of these materials, five different square sizes (i.e., 300 × 300 mm^2^, 400 × 400 mm^2^, 600 × 600 mm^2^, 900 × 900 mm^2^, and 1200 × 1200 mm^2^), which reflect the sizes commonly used on the market, were considered. It should be noted that partition walls were not considered, since they rarely crack before floorings due to the presence of the arch effect (see Section 2.1). Therefore, flooring limit curvatures may be considered as lower bound values with respect to limit curvatures associated with partition walls cracking. However, when flooring cracking does not represent an issue (e.g., in the case of flexible floorings), the same analysis proposed for floorings in this section could be easily extended to various types of partition walls, where the limit curvature shall be determined by analyzing the partition wall behavior (see e.g., [39]). In this case, the limit curvature is the maximum curvature of the partition wall surface in contact with the slab.

According to [31], the strength of ceramic tiles can be expressed by the breaking strength (*S*) obtained by a three-point bending test:

(1) $$S=\frac{FL}{b}\;$$

where *F* is the breaking force, *L* is the span between the supports, and *b* is the width of the specimen. Therefore, the associated bending moment at breaking (*M_u_*) is:

(2) $$M_{u}=\frac{FL}{4}$$

and the limit curvature of the tile ($\chi_{\lim}$) (a plate bent in one direction only) is:

(3) $$\chi_{\lim}=\frac{12\left(1-\nu^{2}\right)M_{u}}{bEs^{3}}=\frac{3\left(1-\nu^{2}\right)S}{Es^{3}}$$

where *E* is the material elastic modulus, ν the Poisson’s ratio, and *s* the thickness of the specimen.

Considering ceramic tiles currently available on the market and setting *E* = 60 GPa and ν = 0.28 [40,41], Equation (3) was used to compute the limit curvatures for the tile sizes studied, which are reported in Table 1. In Table 1, the breaking strengths (*S*) considered are representative values currently available on the market for each specific tile size [28,34,35,36,37].

The strength of marble and granite tiles is usually expressed by the bending strength *f* [31]:

(4) $$f=\frac{3FL}{2bs^{2}}$$

Therefore, the limit curvature can be obtained as:

(5) $$\chi_{\lim}=\frac{12\left(1-\nu^{2}\right)M_{u}}{bEs^{3}}=\frac{2\left(1-\nu^{2}\right)f\;}{Es}$$

The limit curvatures computed for marble tiles (*E* = 123 GPa and ν = 0.25 [29]) are shown in Table 2, whereas Table 3 shows the limit curvatures computed for granite tiles (*E* = 90 GPa and ν = 0.25 [29]); *f* in Table 2 and Table 3 are representative values of the bending strength for the specific tile size considered and available on the market.

The elastic moduli of these materials are extremely variable, in relation to the porosity of the material. Furthermore, stone tiles can be natural or artificial, i.e., obtained with a sintering process that maximizes their mechanical characteristics [28]. There are marble tiles with an elastic modulus between 57 GPa and 123 GPa, while the elastic modulus of ceramic tiles may vary between 40 GPa and 60 GPa. Since the elastic modulus is almost never declared by the manufacturers, in this paper the highest values of *E* found in the literature were conservatively considered.

## 4. Comparison between the Eurocode Limits and the Performance Requirements of the Flooring

In this section, the three member types considered, namely rib and clay pot, composite steel–concrete, and timber beam slabs, are designed to respect the deflection limits provided by the Eurocodes. The maximum curvature of these members is then compared with the limit curvature obtained for the floorings considered, in order to verify the reliability of this approach.

The deflection of a structural member depends on its geometry, mechanical properties, number of spans, type of constraints, and applied loads. Considering uniformly distributed applied loads, the case of a simply supported beam provides the highest maximum deflection. Therefore, this configuration is adopted here, although any different configuration could be analyzed depending on the specific case studied. According to [6], the uniformly distributed load was determined as the sum of the self-weight of the building slab, including flooring, of a load of 2 kN/m^2^, which is the distributed (equivalent) load of partition walls made of hollow clay bricks, and of a variable load of 2 kN/m^2^ or 5 kN/m^2^, which are the variable loads for residential and commercial buildings, respectively. Four values of the span were considered, namely 4 m, 5 m, 6 m, and 7 m. These span values are representative of common values adopted for the slab types studied in this paper. While span values shorter than 4 m would lead to small maximum deflections, which are not likely to impair the integrity of the floor, spans larger than 7 m would lead to excessive slab depth, which would rarely be adopted in practice.

Different concrete classes were considered in the calculations, which provided consistent results regardless of the concrete strength. Indeed, it should be noted that the concrete strength does not play a fundamental role in the definition of the member deflection, where the elastic modulus (not the strength) is the crucial parameter. With increasing the concrete compressive strength, the elastic modulus only slightly increases (e.g., increasing the concrete class from C25/30 to C40/50 leads to an increase of the elastic modulus of only 17% [4]). Furthermore, provided a certain span and applied load, the concrete class affects the height of the designed cross-section, while the maximum deflection (enforced by the Eurocodes) remains the same. In general, an increase in the concrete strength leads to a decrease of the member cross-section height, which, in turn, may determine an increase of the slab deflection. However, high concrete strength is associated with low shrinkage and high concrete tensile strength, which entail for small cracked portions and a consequent decrease of the slab deflection. These contrasting effects (decrease of the cross-section height and high concrete properties) do not generally lead to significant differences in the deflection of slabs with concrete of different strength classes. Therefore, only the results obtained considering a concrete class C25/30 are provided in this paper.

### 4.1. Rib and Clay Pot Slab

**Figure 1:**
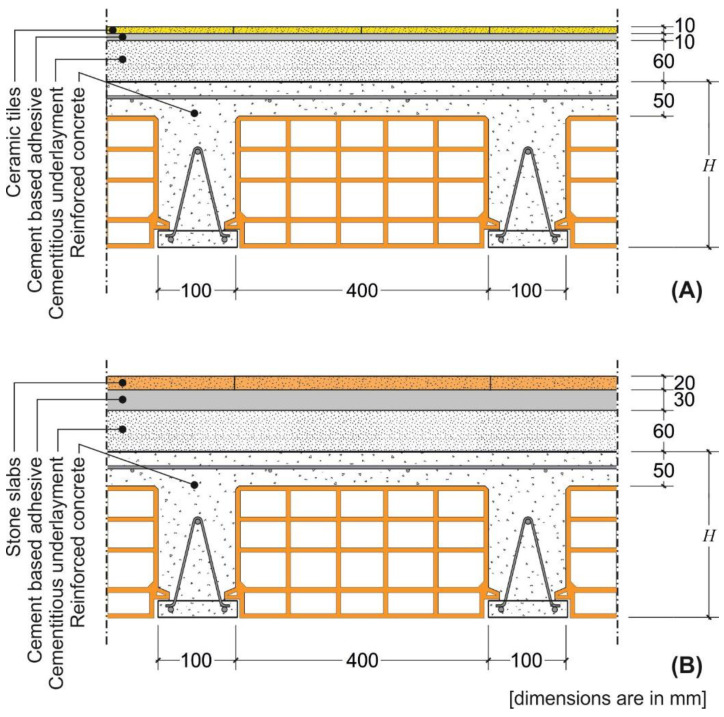
Cross-section of the rib slab with (**A**) ceramic and (**B**) stone floorings.

A representative rib and clay pot slab cross-section with ceramic and stone floorings was considered in this study (Figure 1). The cross-section geometry, which was determined starting from the standardized geometry of the hollow blocks, was kept constant except for the height (H). Provided the applied load associated with the specific flooring and type of building (residential or commercial, see previous section), the minimum value of the height (H) was calculated in order to satisfy the deflection limit enforced by Eurocode 2 (independently of the height of the hollow blocks effectively available on the market). This H value was then considered to verify whether the maximum curvature along the member exceeds the limit values provided in Table 1, Table 2 and Table 3.

The rib and clay pot slabs were designed assuming no contribution of the cementitious underlayment and hollow blocks (Figure 1) to the slab structural response. The underlayment is usually made with a mixture of water, sand, and cement and has a low compressive strength, which can be neglected.

The hollow blocks are employed to decrease the weight of the slab and, unless specific cases where they have low percentage of voids and certain geometrical characteristics [42], do not contribute to the structural response of the slab.

Eurocode 2 allows two alternative verifications for the limit state of deflection, one based on a limit of the span/depth ratio and the other on a limit of the deflection. The deflection limits cannot be directly compared with the limit curvatures of the floorings. Moreover, two distinct limits for the deflection of horizontal members are provided, namely:

- “the appearance and general utility of the structure could be impaired when the calculated sag of a beam, slab or cantilever subjected to quasi-permanent loads exceeds span/250” [4];
- “deflections that could damage adjacent parts of the structure should be limited. For the deflection after construction, span/500 is normally an appropriate limit for quasi-permanent loads” [4].

The presence of two different limits is confusing and deflection values lower than these limits do not always guarantee the absence of damage to the superstructures. In fact, these limits should be verified only with respect to the quasi-permanent load combination, without considering other load combinations associated with service loads (e.g., the characteristic combination) that might affect the construction appearance. However, limiting the deflection verification to the quasi-permanent load combination does not guarantee the integrity of the superstructures under the characteristic load combination, which will certainly occur during the service life of the structure.

The minimum cross-section height (*H*_min_) that satisfies limits (i) or (ii) of Eurocode 2 was determined iteratively by matching the calculated sag with the sag limit value (*w*_max_) (i.e., span/250 or span/500):

*w*(*H*_min_) = *w*_max_ (6)

After assigning the guess value of *H*, the phases of the iterative process were:

1. Determination of the self-weight of the member (slab).
2. Determination of the cross-section cracking moment, where the tensile strength of concrete was computed according to Eurocode 2 [4].
3. Identification of the structural element cracked segment (located at midspan) and of the two symmetrical uncracked segments (located at the supports).
4. Computation of the second moment of area (*J*) of the reinforced concrete cross-section of each segment:

(7) $$J=\overset{}{\underset{A_{c}}{\int }}y^{2}dA_{c}+m\underset{i}{\sum }A_{si}y_{si}^{2}$$

where *A_c_* is the concrete un-cracked area (i.e., the entire cross-sectional area when *J* is associated with a bending moment lower than the cracking moment or concrete compressed area when *J* is associated with a bending moment higher than or equal to the cracking moment), *y* is the vertical distance measured from the cross-section neutral axis, *A_si_* is the *i*-th longitudinal steel cross-sectional area, *y_si_* the vertical distance between the *i*-th longitudinal steel cross-section centroid and the neutral axis, and *m* is the ratio between the steel elastic modulus *E_s_* = 200 GPa [43] and concrete effective elastic modulus *E_ce_*. To account for the long-term behavior of the RC cross-section under the quasi-permanent load combination, *E_ce_* was computed as $E_{ce}=E_{cm}/\left[1+\varphi \left(\infty ,t_{0}\right)\right]$, where *E_cm_* is the concrete elastic modulus at 28 days, and $\varphi \left(\infty ,t_{0}\right)$ is the creep coefficient [4].

5. Determination of the sag *w*(*H*) under the quasi-permanent load.
6. Comparison of the sag *w*(*H*) with the limit *w*_max_ and determination of a new guess value of *H*, until the calculated sag matches the limit value.

**Figure 2:**
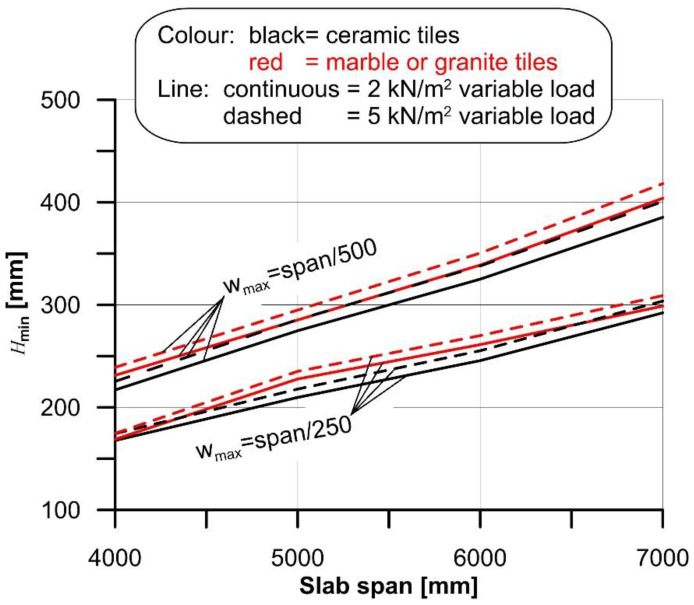
*H*_min_ computed for 4 m, 5 m, 6 m, and 7 m span of the rib and clay pot slab.

The minimum cross-section height (*H*_min_), obtained for the four spans considered in the case of rib and clay pot slab with ceramic and marble or granite tiles and the different variable loads selected, are shown in Figure 2.

Once *H*_min_ was determined, the cross-section curvature at midspan due to the maximum service load, i.e., the maximum curvature χ_max_, was computed and then compared with χ_lim_. Since the flooring is applied when the structure is already bent due to the presence of the permanent load, χ_max_ was computed as the sum of curvature due to long-term permanent load χ_max,*pl*_ and the curvature due to variable load χ_max,*v*_, minus the curvature due to the permanent load acting when the flooring was applied χ_max,*p*_ (i.e., in the absence of concrete creep):

(8) $$\chi_{\max}=\chi_{\max,pl}+\chi_{\max,\nu }-\chi_{\max,p}$$

χ_max,*v*_ and χ_max,*p*_ were computed considering the secant modulus of elasticity of concrete *E_cm_*, whereas χ_max,*pl*_ was computed considering $E_{cm}/\left[1+\varphi \left(\infty ,t_{0}\right)\right]$, where $\varphi \left(\infty ,t_{0}\right)$ is the concrete creep coefficient:

(9) $${\;\chi }_{\max,\nu }=\frac{M_{\max,\nu }}{E_{cm}J}$$

(10) $${\;\chi }_{\max,p}=\frac{M_{\max,p}}{E_{cm}J}$$

(11) $${\;\chi }_{\max,pl}=\frac{M_{\max,pl}}{E_{cm}J}\left[1+\varphi \left(\infty ,t_{0}\right)\right]$$

where *M*_max,*v*_, *M*_max,*p*_, and *M*_max,*pl*_ are the maximum bending moments associated with the variable load, permanent load acting when the flooring was applied, and long-term permanent load, respectively, and *J* is the second moment of area of the cross-section considered. It should be noted that, although the use of this approach to account for the long-term behavior of concrete is only an approximation, it provides conservative results [4,44,45] and a refined and complex analysis (see for instance [46]) would not markedly affect the calculated curvature.

The effect of slab shrinkage was neglected in this paper because it was assumed that, when the flooring was placed, the slab already underwent most of the drying shrinkage and no significant further shrinkage would occur. However, even in those cases where the slab is still undergoing shrinkage when the flooring is applied, shrinkage will not play a significant role in the slab curvature. Shrinkage may induce curvature to RC members due to the eccentricity of the steel longitudinal reinforcement with respect to the cross-section centroid [47]. However, RC members usually have both tension and compression longitudinal steel reinforcement, which limits the effect of shrinkage on the cross-section curvature. Furthermore, the effect of shrinkage on the cross-section curvature is significantly lower than that of creep. As an example, considering the 5 m span slab made by concrete with *f_ck_* = 35 MPa and where the top (or bottom) face is fully constrained, when the deflection limit is set to *L*/500, the corresponding minimum slab height is 275 mm and the curvature induced by shrinkage after 10,000 days (member loaded after 3 days from casting, notional size conservatively assumed equal to 275 mm, RH = 75%) is χ_sh_= 1.345·10^−6^, according to the approach provided by Model Code 2010 [48]. The comparison between this curvature and that obtained for the same cross-section with Equation (8), considering a span *L* = 5 m and a variable load *q* = 2 kN/m^2^, χ_max_ = 1.580·10^−5^, shows that neglecting shrinkage would lead to an underestimation of the cross-section curvature of only 8.5%. This underestimation decreases to 3.5% if the deflection limit is set to *L*/250 and a variable load *q* = 5 kN/m^2^ is considered. These examples—which overestimated the shrinkage contribution to the cross-section curvature, since one side of the cross-section was assumed fully constrained—support the decision of neglecting shrinkage in the computation of the cross-section maximum curvature.

**Figure 3:**
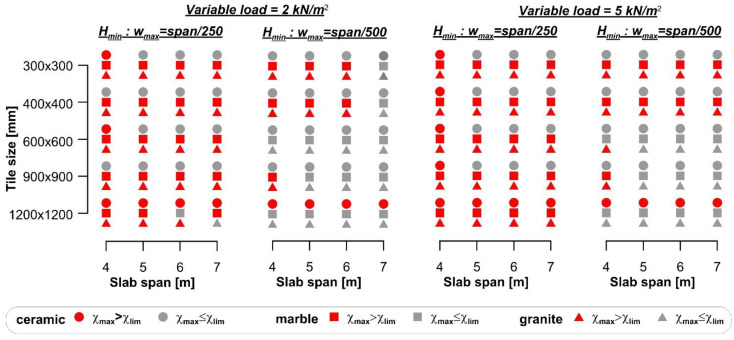
Comparison between the maximum and limit curvatures of floorings for rib and clay pot slabs.

The results of the comparison between the curvature χ_max_ and the flooring limit curvature χ_lim_ are summarized in Figure 3, where red markers indicate cases where the flooring cracks, although the specific Eurocode 2 limit was respected. When the span/250 limit was adopted, flooring cracking occurred in the majority (76.7%) of the cases. However, even when the span/500 limit was adopted, 34.1% of cases led to flooring cracking. The comparison between the maximum curvature (χ_max_) and limit curvature (χ_lim_) showed that damage of the floorings occurred more frequently for small spans than for large spans. This result is due to the use of the deflection limits provided by the Eurocode to design the slab thickness. In the case of a simply supported beam with a parabolic bending moment along its axis, the relationship between the deflection limit provided by the Eurocode was *w*_max_ = *L*/*k*, where *k* is a dimensionless parameter related to the specific structure type, and the maximum curvature χ_max_ can be expressed as:

(12) $$w_{\max}=\frac{L}{k}=\frac{5}{48}\chi_{\max}L^{2}\Rightarrow \chi_{\max}=\frac{48}{5kL}$$

Equation (12) clearly shows that, provided the parameter *k*, the maximum curvature that complies with the Eurocodes decreases for increasing spans. Since the limit curvature is constant (provided a certain type of tyle), a low value of *L* implies higher probability of damage of flooring.

The limit curvature of tiles in Figure 3 depends on their bending stiffness, which was obtained from experimental tests performed according to specific standards and reported in the datasheet. It should be noted that varying the dimension of the tile does not entail for a linear variation of its thickness, whereas the elastic modulus remains constant. Therefore, the limit curvature does not vary linearly with the variation of the tile dimension, which explains why flooring cracking seems independent from the tile dimensions in Figure 3.

### 4.2. Composite Steel–Concrete Slab

The design and requirements for composite steel–concrete slabs are provided by Eurocode 4 [6]. According to it, the maximum deflection of these types of structural member should comply with the limits enforced by Eurocode 3 [49], which, in turn, refers to the national annex. Therefore, the deflection limit span/250 under the characteristic load combination [9] adopted by the Italian code [50] will be considered in this section.

**Figure 4:**
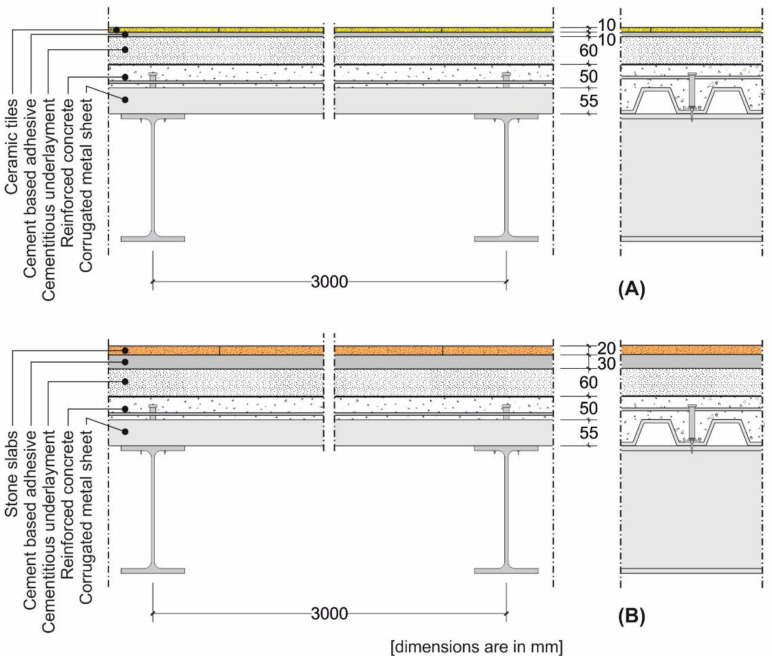
Cross-section of the composite steel–concrete slab with (**A**) ceramic and (**B**) stone floorings.

A representative composite steel–concrete slab cross-section with ceramic and stone floorings was considered in this study (Figure 4).

**Figure 5:**
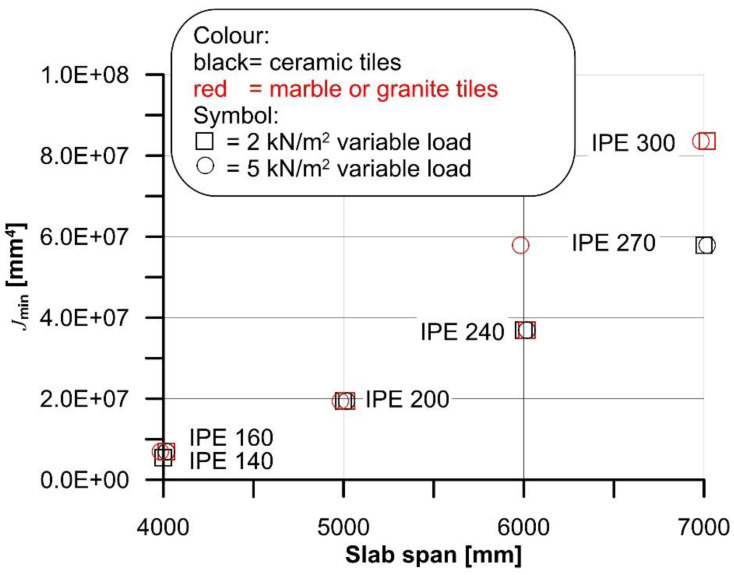
*J*_min_ computed for 4 m, 5 m, 6 m, and 7 m span of the composite steel–concrete slab.

The cross-section geometry, which was determined starting from standardized I-shaped steel beam geometry, was kept constant, except for the second moment of area of the steel beam. Provided the applied load associated with the specific flooring and type of building (residential or commercial), the minimum value of the steel beam second moment of area *J*_min_ was (iteratively), computed to match the deflection limit span/250. The presence of creep of concrete and effective width (*b*_eff_) were taken into account in the calculations, following the procedure proposed in [6]. *J*_min_ obtained for the four spans considered with ceramic and marble or granite tiles and the different variable loads selected are shown in Figure 5.

**Figure 6:**
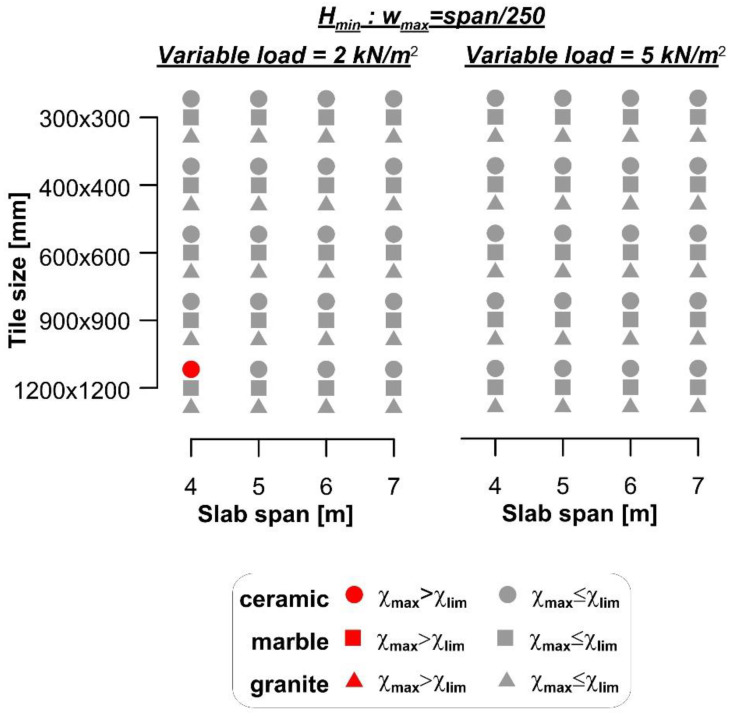
Comparison between the maximum and limit curvatures of floorings for composite steel–concrete slabs.

The results of the comparison between the curvature (χ_max_) and the flooring limit curvature (χ_lim_) are reported in Figure 6, where red markers indicate cases where the flooring cracks. Figure 6 shows that, although the deflection limit span/250 was fulfilled, large ceramic tiles cracked, despite the fact that the adopted steel beams were commercially available products and, therefore, their second moment of area was higher than the minimum required (*J*_min_).

### 4.3. Traditional Slab Made of Timber Beams and Planks

The approach of Eurocode 5 [7] to the deflection analysis of timber structures is complex, since limits on the instant sag (*w*_inst_), final net sag (*w*_net,fin_), and final sag (*w*_fin_) are enforced. For each sag considered, a range of limit values within which the specific sag limit should be determined based on the determined acceptable level of member deformation, is provided. For the sake of simplicity, only the *w*_net,fin_ limit, which ranges between span/250 and span/350 for simply supported beams [7], is considered in this section. However, the same approach described for *w*_net,fin_ can be applied in the case of *w*_inst_ and *w*_fin_.

**Figure 7:**
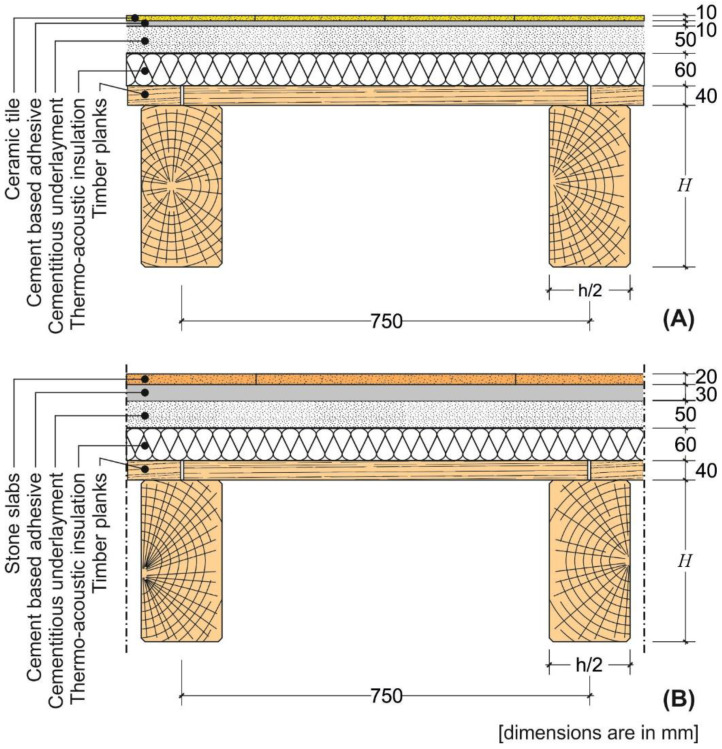
Cross-section of the timber beam and plank slab with (**A**) ceramic and (**B**) stone floorings.

A representative cross-section of a traditional slab made by timber beams and planks with ceramic and stone floorings was considered in this study (Figure 7). Similar to the case of rib slabs (Section 4.1), the cross-section was kept constant, except for the height *H* of the timber beams (joists), which was iteratively computed to match the minimum and maximum limit deflections of the range provided by Eurocode 5 [7], i.e., span/250 and span/350, respectively.

Timber beams develop time-dependent (creep) deformation when subjected to long-term applied loads. Equation (13) was used in this study to compute the final net sag, accounting for the beam creep and shear deformations [7]:

(13) $$w\left(H_{\min}\right)=g\Delta \left(1+k_{\mathrm{def}}\right)+q\Delta \left(1+\psi_{2.1}k_{\mathrm{def}}\right)=w_{\mathrm{net},\mathrm{fin}}$$

(14) $$\Delta =\frac{5}{16}\times \frac{L^{4}}{E_{m}H_{\min}^{4}}+\alpha \frac{L^{2}}{4G_{m}H_{\min}^{2}}$$

where *g* is the permanent load, *q* the variable load, *k*_def_ = 0.6 a deformation factor that accounts for creep deformations, $\psi_{2.1}=0.7$ the factor for quasi-permanent value of a variable action [9], α = 1.2 is the form factor [51], and *E*_m_ = 11.6 GPa and *G*_m_ = 720 MPa are the timber elastic and shear modulus, respectively [52].

**Figure 8:**
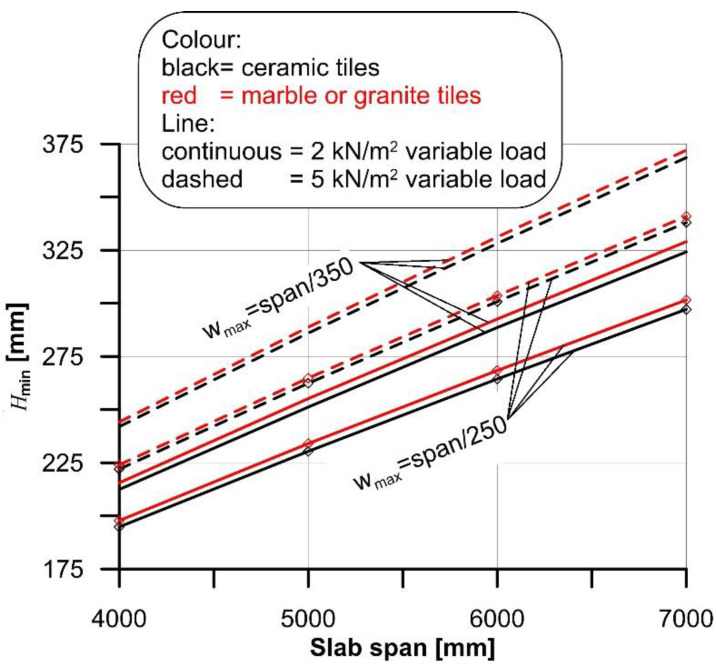
*H*_min_ computed for 4 m, 5 m, 6 m, and 7 m span of the timber slab.

**Figure 9:**
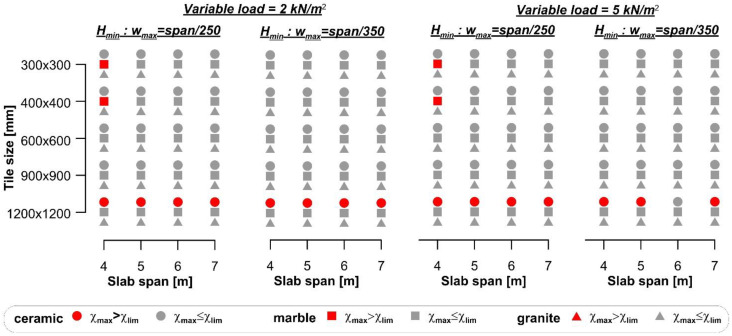
Comparison between the maximum and limit curvatures of floorings for traditional timber beam and plank slabs.

The minimum cross-section height (*H*_min_) obtained for the four spans considered with ceramic and marble or granite tiles and the different variable loads selected, are shown in Figure 8. The results of the comparison between the curvature (χ_max_) and flooring limit curvature (χ_lim_) are reported in Figure 9, where red markers indicate cases where the flooring cracks. Figure 9 shows that ceramic tiles with large size (i.e., 1200 × 1200 mm^2^) cracked in almost all cases (93.8%) considered, whereas 5% of marble tiles and no granite tiles cracked.

### 4.4. Effect of the Underlayment

The analyses described in Section 4.1, Section 4.2 and Section 4.3 were carried out assuming a rigid cementitious underlayment (Figure 1, Figure 4 and Figure 7). To verify the reliability of this assumption, a non-linear finite element (FE) model of a strip 1 m wide and 4 m long of the rib and clay pot slab, designed in Section 4.1, was used to obtain the flooring curvature considering a deformable underlayment. The same analysis was carried out for the composite steel–concrete and timber beam slabs of Section 4.2 and Section 4.3, but the results were not described here for brevity. All models were developed in the FE software Abaqus [53].

Three types of underlayment [54,55,56,57] and three types of flooring, with mechanical properties assumed according to the values provided by [58], were considered in the FE model. The elastic modulus and Poisson’s ratio of the underlayment and floorings modeled are reported in Table 4.

**Figure 10:**
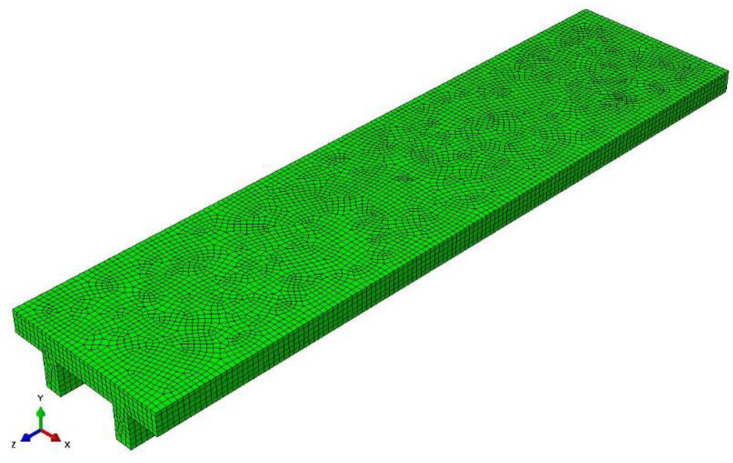
Discretization of the FE model.

The C25/30 concrete was modeled using the concrete damaged plasticity model (CDP) available in Abaqus, whereas the cementitious underlayment, which was assumed to behave as a granular-like soil, was modeled using a linear Drucker–Prager model [53]. The parameters needed for the CDP model were defined following the procedure suggested in [59], which provided a dilation angle ψ = 36° [53], whereas the concrete secant elastic modulus *E_cm_* = 31 GPa provided by the Eurocode 2 for a C25/30 concrete was considered. The underlayment was modeled considering a linear behavior (friction angle φ = 30°) up to the minimum tensile strength required by EN 13813 [58], i.e., 5 MPa, which was followed by a softening behavior to account for possible material failure [60]. The softening branch was defined following the softening curve generally adopted for cohesive materials proposed by [61], which conservatively did not account for compaction of the granular-like underlayment. A 950 N force, which simulates the force induced by the foot of a bookcase, was applied as a uniformly distributed load on a 40 mm diameter circular surface. Different mesh sizes, of either 8-node solid elements or 4-node tetrahedral elements, were used to investigate their effect on the model convergence and time required to obtain the solution. This study resulted in a FE model discretized using 8-node solid elements with approximate dimensions of 20 × 20 × 20 mm^3^ (see Figure 10).

**Figure 11:**
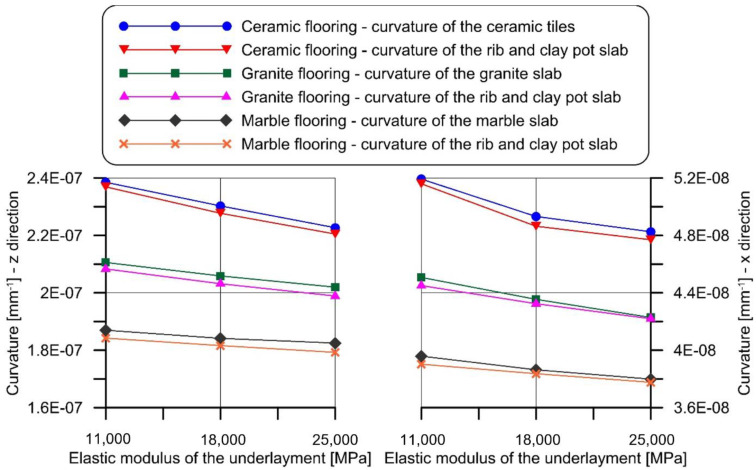
Comparison between the FE model curvatures of the rib and clay pot slab and those of the flooring.

The cross-section deformations, obtained by the FE model, were used to obtain the curvatures of the flooring and of the supporting slab. The results showed that the maximum difference between the curvature of the flooring and of the supporting slab was always lower than 2%, in all the cases considered (Figure 11). This confirms that assuming a rigid underlayment did not significantly affect the result of the analytical procedure adopted in this paper. The same conclusion was obtained for composite steel–concrete and timber beam slabs.

## 5. Discussion

The analysis carried out on the different slab and flooring types showed that the deflection limits provided by the Eurocodes do not always guarantee the integrity of the superstructures. The approach adopted by the Eurocodes could be improved by reducing the maximum displacement allowed. Nevertheless, the study carried out highlights some limitations of the deflection control method, at least in the case of reinforced concrete or rib and clay pot slabs, where the calculation of the deflection is so complex that it is either oversimplified or not performed at all. Indeed, under service conditions, the simultaneous presence of cracked and non-cracked areas, which are also affected by time-dependent (viscous) phenomena and oligo-cyclic variable loads, makes correctly computing the member deflection extremely difficult [62]. The temporal evolution of the behavior of a reinforced concrete cracked section is already a quite complex problem (see for instance [43]). If then the effect of tension stiffening has to be accounted for, together with its temporal evolution due to creep, and the integration of all these phenomena over the whole length of the beam has to be carried out, the problem becomes unreasonable for a professional engineer, who must necessarily maintain a correct cost-benefit ratio in the design work. Design standards should, as far as possible, provide rules that respect this ratio. To overcome the issues associated with the deflection control method, a curvature control method, as a verification method of the horizontal member deflection under service loads, is proposed and discussed in the next sections.

### 5.1. Curvature Control Method

In this section, the possibility of using the criterion adopted to verify the reliability of the Eurocode deflection limits, i.e., the comparison between the maximum member curvature (χ_max_) and limit curvature of the specific finishing elements (χ_lim_) (or conservatively of the stiffest finishing elements), is discussed.

This method, referred to as curvature control method, is more reliable than the deflection control method currently provided by the Eurocodes, since it allows a direct comparison with the limit curvature of the finishing elements. Furthermore, computing the maximum member curvature is simpler than computing the corresponding maximum deflection. Indeed, the member deflection depends on several factors, including the load type and distribution, member static scheme, and flexural stiffness of the various elements that compose it, which determines the need of a double integration of the curvature along the entire member extent to compute its maximum deflection. This calculation becomes particularly complex when dealing with cracked concrete, since, in this case, the flexural stiffness varies along the member.

The curvature control method, unlike the deflection control method, requires a local analysis at the cross-section with the highest bending moment, independent of the member static scheme. The curvature is independent of the member span, which is required only to define the maximum service bending moment. Therefore, provided the maximum service bending moment and cross-section bending stiffness, the cross-section curvature can be simply computed without any integration along the member axis. For those cases where a deflection control is still required (e.g., to guarantee adequate drainage), the member maximum deflection could be computed by double integration of the limit curvature, which could be conservatively assumed constant along the longitudinal axis of the member considered. This computation would not require the knowledge of the curvature along the member and provide conservative results.

**Figure 12:**
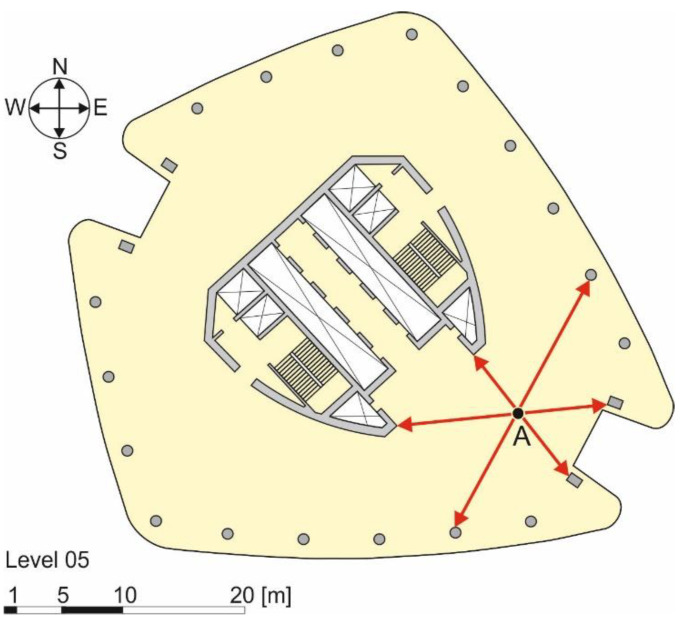
Plan of the fifth floor of the Generali Tower (by Zaha Hadid Architects) in Milan.

As an example of the advantages of adopting a curvature control rather than a deflection control method, the case of the Generali Tower, a 44-story building by Zaha Hadid Architects, built in Milan (Italy), between 2014 and 2017, is examined. The building structure consists of columns and a central core supporting structural slabs, all made of reinforced concrete. When performing the deflection control in point A of Figure 12, according to Eurocode 2 [4], many different spans (indicated with arrows in Figure 12) can be considered, each providing a specific deflection limit. Although considering the shortest span would lead to the smallest maximum deflection possible, this value could be either applied to the entire slab or only to the span selected, while other values could be computed for the other spans. By performing the deflection control based on the curvature limit, this issue does not exist, since a unique value of the maximum curvature under service loads is associated to point A. A simple finite element model of the building (needed due to the complexity of the structure) would be sufficient to determine the cross-sections with the highest applied stress and corresponding bending moments in two orthogonal directions, which can be used to compute the cross-section maximum curvature.

### 5.2. Deflection Control Based on the Limit Curvature

Assuming that the flexural stiffness along the member longitudinal axis is constant and equal to the flexural stiffness of the cross-section associated with the maximum bending moment, the limit curvature could be used to conservatively estimate the member maximum deflection, such that no damage occurs to the superstructures. Considering the case of a generic simply supported ribbed slab where the shear deformability is neglected under service loads, the maximum curvature can be computed as the sum of the curvature due to permanent loads (indicated with the subscript *g*) and curvature due to variable loads (indicated with the subscript *q*):

(15) $$\chi_{\lim}\leq \frac{M_{\max,g}}{{\left(EJ\right)}_{g}}+\frac{M_{\max,q}}{{\left(EJ\right)}_{q}}$$

where (*EJ*)*_g_* and (*EJ*)*_q_* are the cross-section flexural stiffness under the permanent and variable loads, respectively, which may differ due to the cross-section applied stress and short- or long-term material properties considered. Equation (15) provides a simple solution to compute the cross-section curvature. Flexural stiffnesses under permanent and variable loads were defined separately to properly account for concrete creep, which is associated with the permanent load. Following the approach of Eurocode 2 [4], the flexural stiffnesses under permanent loads can be defined as:

(16) $${\left(EJ\right)}_{g}=E_{cm}J/\left[1+\varphi \left(t,t_{0}\right)\right]$$

where $\varphi \left(\infty ,t_{0}\right)$ ≈ 2 and *J* is the cross section second moment of area computed with Equation (7). Although more refined approaches can be adopted (see e.g., [46]), they would require complex numerical solutions that do not appear suitable for current practice applications. Note that creep plays a major role in the definition of the limit curvature and should not be neglected. As an example, in the case of rib and clay pot slabs with span varying from 4 m to 7 m (see Figure 2), neglecting creep (i.e., setting $\varphi \left(\infty ,t_{0}\right)=0$) would result in an average slab maximum curvature (χ_max_) 61% and 40% lower than that obtained when considering creep when the variable load is *q* = 2 kN/m^2^ and *q* = 5 kN/m^2^, respectively.

Assuming uniformly distributed applied loads, Equation (15) can be rewritten as:

(17) $$\chi_{\lim}\leq \frac{gL^{2}}{8{\left(EJ\right)}_{g}}+\frac{qL^{2}}{8{\left(EJ\right)}_{q}}=\frac{L^{2}}{8}\left[\frac{g}{{\left(EJ\right)}_{g}}+\frac{q}{{\left(EJ\right)}_{q}}]\Rightarrow [\frac{g}{{\left(EJ\right)}_{g}}+\frac{q}{{\left(EJ\right)}_{q}}\right]\leq \frac{8\chi_{\lim}}{L^{2}}$$

Similarly, the maximum vertical displacement (*w*_max_) of the same generic simply supported ribbed slab can be obtained as the sum of the displacement induced by the permanent loads and displacement induced by the variable loads:

(18) $$w_{\max}=\frac{5}{384}\left[\frac{gL^{4}}{{\left(EJ\right)}_{g}}+\frac{qL^{4}}{{\left(EJ\right)}_{q}}\right]=\frac{5L^{4}}{384}\left[\frac{g}{{\left(EJ\right)}_{g}}+\frac{q}{{\left(EJ\right)}_{q}}\right]$$

*w*_max_ can be expressed as a function of the limit curvature by substituting Equation (17) into Equation (18):

(19) $$w_{\max}\leq \frac{5\chi_{\lim}L^{2}}{48}$$

According to the Eurocodes, the maximum deflection should respect the inequality in Equation (20) (see Section 4.1):

(20) $$w_{\max}\leq \frac{L}{k}$$

where *k* is a dimensionless parameter related to the specific structure type. Therefore, rearranging Equation (20) and substituting *w*_max_ provided by Equation (19) into it, *k* can be expressed as a function of the limit curvature:

(21) $$k\leq \frac{48}{5\chi_{\lim}L}$$

Equation (21) shows that *k* is independent of the flexural stiffness of the element and applied loads, which makes it suitable for applications to any type of structure. Furthermore, the ratio 48/5 in Equation (21) accounts for the specific applied load distribution (this ratio is equal to 12 in the case of a simply supported beam with a concentrated load at midspan), whereas the approach adopted by the Eurocodes [i.e., Equation (20)] is independent from it. If a check based on the curvature control method is adopted, the structural scheme of the slab would not affect the results.

These considerations indicate that the approach adopted by the Eurocodes, based on the vertical displacement limit, may lead to uncertainties in the evaluation of the maximum displacement allowed and provide non-conservative results for certain slab configurations, whereas the approach based on the curvature control method appears more rapid and reliable. Moreover, adopting the curvature control method would allow us to define a general limit curvature for all types of flooring that would work as a minimum product performance target for the manufacturers and, at the same time, guarantee the absence of cracking in the floorings. Finally, the curvature control method could be also conveniently applied in specific problems associated with the use of innovative technologies that are emerging in the world of construction, such as the case of bridge slabs reinforced with glass fiber-reinforced polymer (GFRP) bars. In this case, the slab design is controlled by the deformability (rather than by the strength), which should be limited to ensure the integrity of the asphalt pavement under the characteristic load combination [10]. Therefore, a simple comparison between the maximum curvature allowed for the asphalt and corresponding slab curvature would be sufficient to verify the slab deformability.

## 6. Conclusions

This paper analyzed the approach provided by the Eurocodes to limit the deflection of horizontal structural members and, in turn, guarantee the integrity of the superstructures. Different types of horizontal member, namely rib and clay pot (or hollow block), composite steel–concrete, and timber beam slabs were designed to respect the deflection limit enforced by the Eurocodes. The maximum curvature of these members was compared with the limit curvatures of various types of flooring to verify the occurrence of damage. The results obtained allowed for drawing the following conclusions:

- The deflection limit method adopted by the Eurocodes is complex and does not always guarantee the absence of damage to the floorings. Although the rules enforced by the Eurocodes for a reinforced concrete or a rib and clay pot slab were respected (except for tension stiffening that was neglected), up to 76.7% of the ceramic, marble, and granite floorings cracked. Furthermore, when dealing with a reinforced concrete or a rib and clay pot slab, the Eurocode 2 approach requires taking into account cracking, concrete creep, and tension stiffening, which make it extremely complex and hardly applicable for a professional engineer.
- The curvature control method is much simpler than the deflection control method adopted by the Eurocodes, since a direct verification on the curvature limits is performed. The curvature control method only requires a cross-section analysis, whereas the deflection control method requires the integration of curvature along the entire member axis.
- The curvature control method considers the constraint of the slab by computing the maximum curvature from the maximum service bending moment, calculated considering geometry, constraints, and intended use of the slab. Similarly, the limit imposed to the displacement in the deflection limit method seems independent from the constraints acting on the slab (it depends just on the span), which are accounted for in the computation of the maximum deflection.
- The curvature control method would allow for defining a general limit curvature value for floorings that could be adopted as minimum performance level in standards and would be able to guarantee the absence of flooring cracking. Furthermore, it appears promising for applications to specific problems arising with the use of innovative technologies, as in the case of bridge slabs reinforced with GFRP bars in which the design is controlled by the slab deformability rather than by its strength.

In conclusion, this research tries, through an alternative proposal, to open the discussion on the theme of deflection control in horizontal members, which is traditionally considered well-established, although it presents unsolved issues.

## Figures and Tables

**Table 1:** Limit curvatures of ceramic tiles.

Size [mm]	*s* [mm]	*S* [N]	$\chi_{lim}\;[10^{-5}\;{\mathrm{mm}}^{-1}]$
300 × 300	9.5	750	4.03
400 × 400	9.5	900	4.84
600 × 600	10	900	4.15
900 × 900	10	1000	4.61
1200 × 1200	20	1200	6.91

**Table 2:** Limit curvatures of marble tiles.

Size [mm]	*s* [mm]	*f* [MPa]	$\chi_{lim}\;[10^{-5}\;{\mathrm{mm}}^{-1}]$
300 × 300	20	12	0.85
400 × 400	20	12	0.85
600 × 600	20	35	2.49
900 × 900	30	35	1.66
1200 × 1200	30	50	2.37

**Table 3:** Limit curvatures of granite tiles.

Size [mm]	*s* [mm]	*f* [MPa]	$\chi_{lim}\;[10^{-5}\;{\mathrm{mm}}^{-1}]$
300 × 300	20	12	1.18
400 × 400	20	12	1.18
600 × 600	20	25	2.47
900 × 900	30	25	1.64
1200 × 1200	30	35	2.30

**Table 4:** Mechanical properties of underlayment and floorings modeled.

Material	Elastic Modulus [GPa]	Poisson’s Ratio
Concrete	32	0.20
Underlayment 1 (cementitious)	11	0.20
Underlayment 2 (cementitious)	18	0.20
Underlayment 3 (cementitious)	25	0.20
Flooring: ceramic tiles	60	0.28
Flooring: marble tiles	132	0.25
Flooring: granite tiles	90	0.25

## Data Availability

The data presented in this study are available on request from the corresponding author.
